# Optimizing nutrient solution for vegetative growth of *Dendrobium* Tubtim Siam and *Phalaenopsis* Taisuco Swan through plant tissue nutrient balance estimation

**DOI:** 10.1186/s12870-024-04931-x

**Published:** 2024-04-13

**Authors:** Milton G. Costa, Cibele Mantovani, Renato de Mello Prado

**Affiliations:** Faculty of Agricultural and Veterinarian Sciences, Department of Agricultural Production Sciences, São Paulo State University (UNESP), Via de acesso Prof. Paulo Donato Castellane, Jaboticabal, São Paulo, 14884900 Brazil

**Keywords:** *Dendrobium* Tubtim Siam, *Phalaenopsis* Taisuco Swan, Plant nutrition, Nutritional balance, Orchidaceae

## Abstract

**Background:**

Orchids are grown without soil in many regions of the world, but there is a lack of studies to define the balanced and adequate nutrient solution for their cultivation, mainly in the vegetative growth phase. Therefore, this paper aims to evaluate the optimal concentration of the nutrient solution based on the proposal by Hoagland and Arnon (1950) in the vegetative growth phase capable of increasing the nutrient contents, growth, and dry matter production of *Dendrobium* Tubtim Siam and *Phalaenopsis* Taisuco Swan. In addition, this paper aims to estimate a new nutrient solution from the optimal nutrient contents in the dry matter of these orchid species to be used in the vegetative growth phase.

**Results:**

Nutrient contents, growth, and dry matter production increased as the nutrient solution concentration increased up to an average concentration of 62 and 77% for *D.* Tubtim Siam and *P.* Taisuco Swan, respectively. We found that the Hoagland and Arnon solution presented a group of nutrients with concentrations above the requirement for *P.* Taisuco Swan (nitrogen, phosphor, calcium, and sulfur) and *D.* Tubtim Siam (phosphor, calcium, magnesium, and sulfur), while other nutrients in the solution did not meet the nutritional demand of these orchid species, inducing nutritional imbalance in the vegetative growth phase.

**Conclusion:**

We conclude that using a balanced nutrient solution created specifically for each orchid species in vegetative growth might favor their sustainable cultivation by optimizing the use of nutrients in the growing medium.

**Supplementary Information:**

The online version contains supplementary material available at 10.1186/s12870-024-04931-x.

## Introduction

Orchids are abundant in the world’s humid tropics, accounting for a large share of the global floricultural trade both as cut flowers and as potted plants, among which 85% are *Dendrobium* species and 15% *Phalaenopsis* and *Cymbidium* species [1]. *Dendrobium* and *Phalaenopsis* orchids have characteristics typical of crassulacean acid metabolism (CAM) plants, such as leaf succulence, nocturnal CO_2_ fixation, and inverted stomatal rhythm [2], having low water losses [3]. However, little is understood regarding the nutritional management of orchids and studies in the literature are still incipient [4–9], mainly in the vegetative growth phase. Most studies related to orchid nutrition focus on the interaction with microorganisms [10–14]. Understanding and optimizing the nutrient requirements of orchids are vital for their successful cultivation and to meet the demands of the orchid industry.

The results of studies on the nutritional management of orchids are diverse, as these are plants capable of adapting to different substrates and different fertilization conditions [15–17]. However, most substrates used in orchid cultivation have low nutrient availability [18, 19], reducing the potential for plant growth. Thus, with the domestication of orchids and large-scale production seeking precociousness, it becomes essential to supply nutrients in an adequate and balanced way [20].

When it is intended to cultivate orchids under artificial conditions, most producers and collectors use mineral fertilization based on other agricultural crops, without meeting the specific nutritional needs of each species. In addition, studies are conducted without balancing the nutrients present in the fertilizer sources used [21, 22]. Thus, studies for the proper nutrition of orchids are necessary, as there is the option of soilless cultivation with inert substrates, which is expanding worldwide. It is necessary to use a nutrient solution for this type of cultivation, which is an excellent alternative, especially for the production of flowers [23].

The nutrient solution proposed by Hoagland and Arnon [24] is the most used in plant nutrition research, forming the basis for the formulation of many nutrient solutions [25]. It is important to emphasize that this nutrient solution has a high concentration of salts in solution, which may impair the growth of some species that are sensitive to salinity [26]. Impairments caused by excess salt in orchids affect root development and may vary according with plant species [27], as there are reports in *Phalaenopsis* showing that the increase in salinity did not affect flower growth [28]. However, another study using a nutrient solution with high salinity, that is, with 1.5 or 2.0 fold the concentration of the standard solution, impaired the growth of *Doritaenopsis* ‘Tinny Tender’ orchids and deteriorated flower quality [29]. Thus, it is important to adjust the ionic strength of the nutrient solution to adequate levels for optimal plant growth [25].

For the Orchidaceae family, the vegetative growth phase holds great importance for floriculturists, as it is during this phase that plants are cultivated in floriculture settings. A recent study conducted with Phalaenopsis indicated that vegetative characteristics can predict the quality of flowering, suggesting that the quantity of floral stems and the number of flowers and buds are related to the number of leaves and vegetative biomass [30]. Subsequently, when the flowering stage begins, they are marketed. Therefore, understanding the ideal nutrient solution for this phase is essential for the success of orchid floriculture, ensuring plants free from nutritional disorders and exhibiting high performance for commercial purposes, thus, proper nutrient management enables the achievement of an economically successful crop [31].

The lack of a balanced nutrient solution with the adequate ionic strength to meet the specific nutritional needs of each species of *Dendrobium* tubtim Siam and *Phalaenopsis* taisuco Swan is a concern [31], since the cultivation of these ornamental plants using empirical forms of nutrient solution (without scientific foundation) may be compromising its optimal growth and development [31]. By evaluating variables such as plant height, pseudobulb diameter, leaf length and diameter, number of leaves, number of pseudobulbs, leaf area, and dry matter production, we can gain valuable insights into the response of these orchids to the nutrient solution. These variables provide quantitative measurements that help us understand the overall growth performance and nutritional status of the plants. For example, the study by Jiménez-Peña et al. [32] found that the Steiner solution increased leaf area, total fresh weight, and total dry weight in *Laelia autumnalis* compared to the control [32]. Similarly, Rodrigues et al. [20] found that the combined application of mineral and organic fertilizers resulted in higher dry matter production in orchid seedlings compared to the isolated application of each fertilizer [20]. These findings suggest that the choice of nutrient solution can significantly impact leaf area and dry matter production in orchids.

In this scenario, it becomes evident the problem of the lack of a specific nutrient solution for the growth phase of the orchid, aiming to understand whether the concentration of salts in the nutrient solution can affect growth in a similar way among orchid species. It is possible to obtain a balanced nutrient solution for different orchid species by estimating the nutrient concentration in the dry matter of plants, following the criteria formulated by Martinez and Clemente [33]. It is possible that the relationships between nutrients in this specific nutrient solution for orchids are different from those found in the nutrient solution proposed by Hoagland and Arnon [24], which is commonly used for different species [31].

In order to improve the understanding of the nutritional management of orchids, it is necessary to evaluate the following hypotheses: (i) the use of the nutrient solution proposed by Hoagland and Arnon [24] in diluted concentrations, depending on the ionic strength, increases nutrient contents, improving the growth and dry matter production of orchid plants in the vegetative growth phase and, if proven; (ii) the nutrient concentration in the nutrient solution is adequate in the proportions estimated by Martinez and Clemente [33], to sustain the nutritional demand of *D.* Tubtim Siam and *P.* Taisuco Swan. Our study aims to evaluate the optimal concentration of the nutrient solution proposed by Hoagland and Arnon [24] able to increase the nutrient content, growth, and dry matter production of *D.* Tubtim Siam and *P.* Taisuco Swan plants during the vegetative growth phase, and to estimate a new nutrient solution from the optimum content of nutrients in the dry matter of these plants.

## Results

### Green color index, nutrient contents, biometry, and biomass production of *Dendrobium* Tubtim Siam

**Fig. 1:**
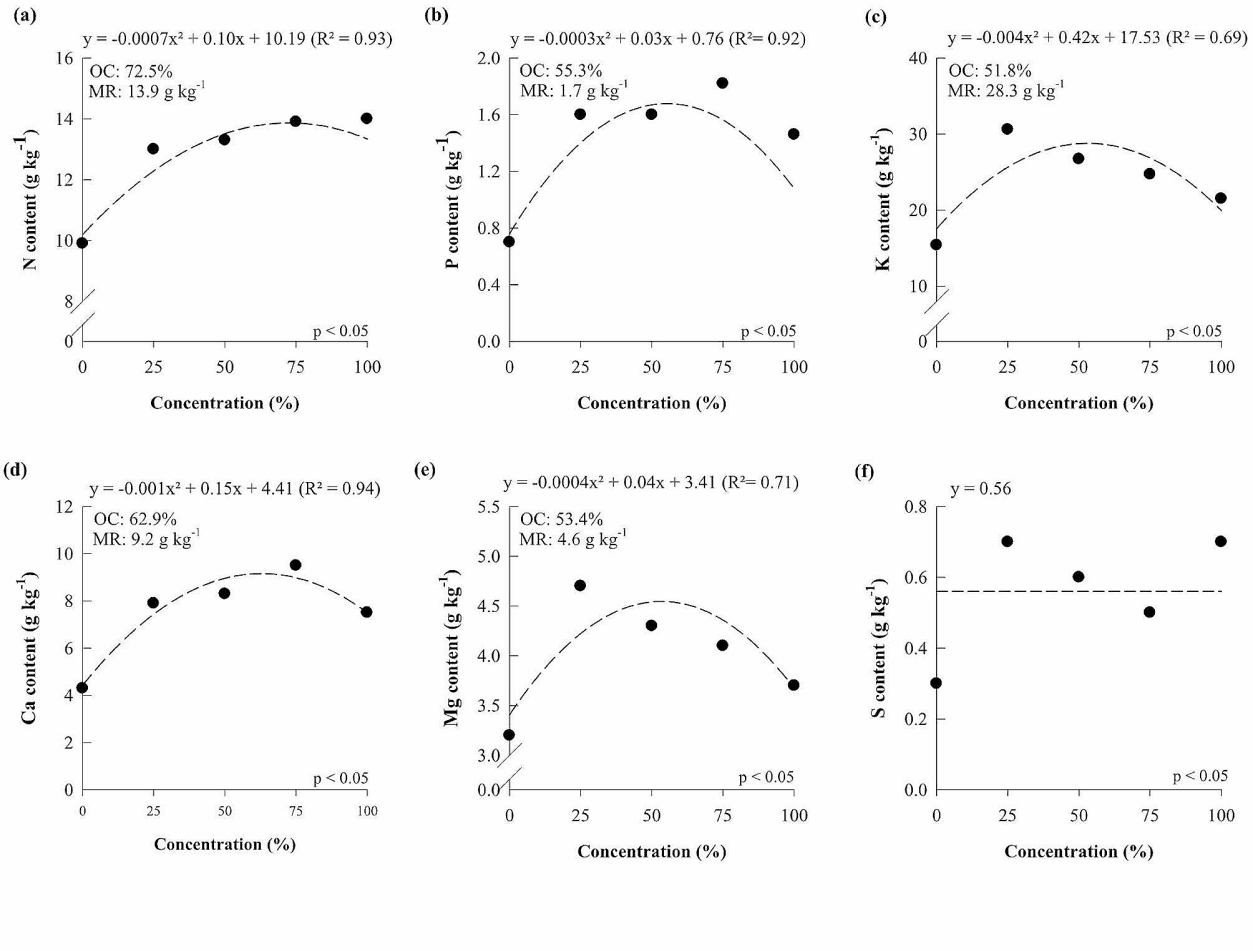
N (**a**), P (**b**), K (**c**), Ca (**d**), Mg (**e**), and S (**f**) content of *Dendrobium Tubtim* Siam grown under different nutrient solution concentrations. OC: optimal concentration; MR: maximum response

Nitrogen (N), phosphor (P), potassium (K), calcium (Ca), and magnesium (Mg) contents in *D.* Tubtim Siam increased as the concentration of the Hoagland and Arnon solution increased, reaching the maximum point at 72.5, 55.33, 51.76, 62.88, and 53.38%, which resulted in contents of 13.87, 1.68, 28.25, 9.16, and 4.55 g kg^-1^, respectively (Fig. 1). For the sulfur (S) content, there was no significant response for the different concentrations of the solution by Hoagland and Arnon.

Boron (B), copper (Cu), iron (Fe), manganese (Mn), and zinc (Zn) contents also increased as the concentration of the Hoagland and Arnon solution increased, reaching the maximum point at 86.53, 68.33, 65.19, 59.18, and 68.52%, which resulted in contents of 35.51, 4.90, 167.63, 295.85, and 201.89 mg kg^-1^, respectively.

The increase in the concentration of the nutrient solution increased the green color index (GCI), plant height, number of leaves, number of pseudobulbs, pseudobulb diameter, and dry matter of *D.* Tubtim Siam, reaching the maximum point at 95.86, 53.67, 42.88, 56.25, 45.95, and 56%, which resulted in 19.0, 135.7 mm, 12.9, 3.5, 16.23 mm, and 1.72 g, respectively (Fig. 3). *D.* Tubtim Siam plants did not respond significantly to the increase in nutrient solution concentration for the variable leaf area (Fig. 3d).

### Green color index, nutrient contents, biometry, and biomass production of *Phalaenopsis Taisuco* Swan

N, P, K, Ca, Mg, and S contents in *P. Taisuco* Swan increased as the nutrient solution concentration increased, reaching the maximum point at 80.7, 89.3, 75.8, 72.5, 50.5, and 75.0%, which resulted in contents of 28.5, 2.6, 54.8, 13.8, 3.8, and 2.3 g kg^-1^, respectively (Fig. 4).

B, Cu, Fe, Mn, and Zn contents also increased as the nutrient solution concentration increased, reaching the maximum point at concentrations of > 100, 84.2, 59.8, 61.3, and 69.9%, which resulted in contents of 77.0, 4.0, 175.7, 342.8, and 206.7 mg kg^-1^, respectively (Fig. 5).

The GCI, number of leaves, length of the largest leaf, diameter of the largest leaf, and dry matter increased as the nutrient solution concentration increased, reaching the maximum point at the concentrations of 112.0, 57.6, 63.95, 56.0, and 57.0%, which resulted in 20.78, 4.44, 194.1 mm, 55.0 mm, and 1.31 g, respectively (Fig. 6). However, there was no significant response for leaf area to increases in the nutrient solution concentration for *P. Taisuco* Swan (Fig. 6c).

### Estimating a new nutrient solution from the optimum contents for growing *Dendrobium* Tubtim Siam and *Phalaenopsis* Taisuco Swan

Mathematical models adjusted from the nutrient contents in the dry matter of *D.* Tubtim Siam and *P.* Taisuco Swan and the general gas equation allowed estimating the ideal nutrient concentrations in the nutrient solution. The N concentration proposed for the nutrient solution was 14.6 and 16.2 mM for *D.* Tubtim Siam and *P.* Taisuco Swan, respectively, while the concentration proposed by Hoagland and Arnon is 15 mM (Table 1).

**Table 1:** Recommended nutrient concentrations in the nutrient solution proposed by Hoagland and Arnon and nutrient concentrations estimated from the optimal nutrient content in the dry matter for *Dendrobium Tubtim* Siam and *Phalaenopsis Taisuco* Swan

Nutrients	Nutrient solution proposed by Hoagland & Arnon (1950)	Nutrient solutions proposed from the optimal concentrations of nutrients	
		Dendrobium Tubtim Siam	Phalaenopsis Taisuco Swan
mM			
N	15.00	14.63	16.19
P	1.00	0.80	0.65
K	6.00	10.68	11.16
Ca	5.00	3.38	2.73
Mg	2.00	2.77	1.25
S	2.00	0.26	0.57
µM			
B	46.30	48.53	56.65
Cu	0.30	1.14	0.50
Fe	17.92	44.35	25.04
Mn	9.10	79.56	49.66
Zn	0.70	45.63	25.16
TIC (mM)	31.07	32.73	32.73
EC (mS cm^− 1^)	2.17	2.28	2.28
Ψ_o_ (Mpa)	0.0706	0.0743	0.0743

TIC: total ion concentration; EC: electric conductivity; Ψ_o_: osmotic potential

The P concentration in the nutrient solution was estimated at 0.8 and 0.65 mM for *D.* Tubtim Siam and *P.* Taisuco Swan, respectively, while the concentration proposed by Hoagland and Arnon is 1.0 mM (Table 1). The K concentration was estimated at 10.7 and 11.2 mM for *D.* Tubtim Siam and *P.* Taisuco Swan, respectively, compared to 6.0 mM proposed by Hoagland and Arnon (Table 1).

The Ca concentration of 3.4 and 2.7 mM proposed for the nutrient solution for *D.* Tubtim Siam and *P.* Taisuco Swan, respectively, was lower compared to the concentration of 5.0 mM proposed by Hoagland and Arnon (Table 1). For Mg, there was an indication of a concentration of 2.8 and 1.3 mM in the nutrient solution for *D.* Tubtim Siam and *P.* Taisuco Swan, respectively, while the concentration indicated by Hoagland and Arnon is 2 mM (Table 1). The greatest difference in the recommended concentrations was verified for S, with a recommendation of 0.26 and 0.57 mM being estimated for *D.* Tubtim Siam and *P.* Taisuco Swan, respectively, while the recommendation of Hoagland and Arnon indicates a concentration of 2 mM (Table 1).

The estimated B concentration in the nutrient solution was estimated at 48.5 and 56.7 µM for *D.* Tubtim Siam and *P.* Taisuco Swan, while a concentration of approximately 46.3 µM was recommended by Hoagland and Arnon (Table 1). For Cu, the concentration of 1.1 and 0.5 µM was estimated for *D.* Tubtim Siam and *P.* Taisuco Swan, respectively (Table 1).

The Fe concentrations in the nutrient solution were estimated at 44.4 and 25.0 µM for *D.* Tubtim Siam and *P.* Taisuco Swan, respectively, being lower than those recommended by Hoagland and Arnon, 89.6 µM (Table 1). The recommendations for Mn and Zn were higher than those indicated by Hoagland and Arnon, 9.1 and 0.7 µM, respectively, as the recommendation of 97.6 µM of Mn and 45.6 µM of Zn were estimated for *D.* Tubtim Siam and 49.7 µM of Mn and 25.2 µM of Zn were estimated for *P.* Taisuco Swan (Table 1).

The N:P, N:Ca, N:S, P:Ca, P:S, K:Ca, K:Mg, K:S, Ca:S, and Mg:S ratios increased and N:K, N:Mg, P:K, P:Mg, and Ca:Mg ratios decreased for *D.* Tubtim Siam in relation to the solution proposed by Hoagland and Arnon (Table 2). For *P.* Taisuco Swan, there was an increase of N:P, N:Ca, N:Mg, N:S, P:Ca, P:Mg, P:S, K:Ca, K: Mg, K:S, Ca:S, and Mg:S ratios and a decrease of N:K, P:K, and Ca:Mg ratios compared to the nutrient solution proposed by Hoagland and Arnon (Table 2) .

**Table 2:** Ratios between macronutrients in the solutions of *Dendrobium Tubtim* Siam and *Phalaenopsis Taisuco* Swan and the solution by Hoagland and Arnon

Ratios	Nutrient solution proposed by Hoagland & Aenon (1950)	Nutrient solutions proposed from the optimal concentrations of nutrients	
		Dendrobium Tubtim Siam	Phalaenopsis Taisuco Swan
N:P	15.00	18.26	24.74
N:K	2.50	1.37	1.45
N:Ca	3.00	4.33	5.92
N:Mg	7.50	5.29	12.91
N:S	7.50	56.70	28.31
P:K	0.17	0.08	0.06
P:Ca	0.20	0.24	0.24
P:Mg	0.50	0.29	0.52
P:S	0.50	3.11	1.14
K:Ca	1.20	3.16	4.08
K:Mg	3.00	3.86	8.90
K:S	3.00	41.37	19.51
Ca:Mg	2.50	1.22	2.18
Ca:S	2.50	13.09	4.78
Mg:S	1.00	10.72	2.19

For micronutrients, there was an increase in the Cu:Fe ratio and a decrease in B:Cu, B:Fe, B:Mn, B:Zn, Cu:Mn, Cu:Zn, Fe:Mn, Fe:Zn, and Mn:Zn ratios for two orchid species studied compared with the solution proposed by Hoagland and Arnon (Table 3).

**Table 3:** Ratios between macronutrients in the solutions of *Dendrobium Tubtim* Siam and *Phalaenopsis Taisuco* Swan and the solution by Hoagland and Arnon

Ratios	Nutrient solution proposed by Hoagland & Aenon (1950)	Nutrient solutions proposed from the optimal concentrations of nutrients	
		Dendrobium Tubtim Siam	Phalaenopsis Taisuco Swan
B:Cu	154.33	42.60	113.82
B:Fe	2.58	1.09	2.26
B:Mn	5.09	0.61	1.14
B:Zn	66.14	1.06	2.25
Cu:Fe	0.017	0.03	0.02
Cu:Mn	0.03	0.01	0.01
Cu:Zn	0.43	0.02	0.02
Fe:Mn	1.97	0.56	0.50
Fe:Zn	25.60	0.97	1.00
Mn:Zn	13.00	1.74	1.97

## Discussion

**Fig. 2:**
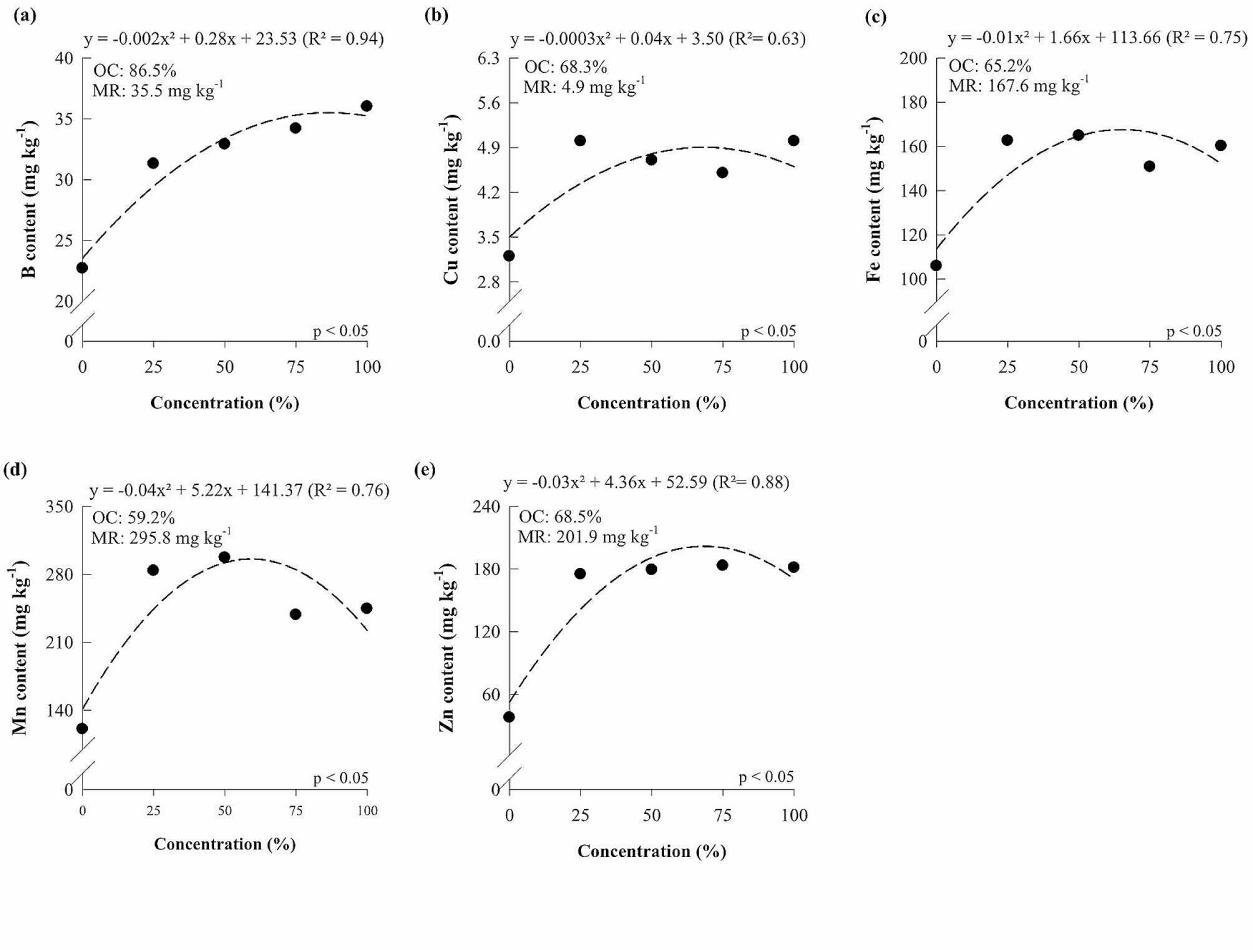
B (**a**), Cu (**b**), Fe (**c**), Mn (**d**) and Zn (**e**) content of *Dendrobium Tubtim* Siam cultivated under different nutrient solution concentrations. OC: optimal concentration; MR: maximum response

**Fig. 3:**
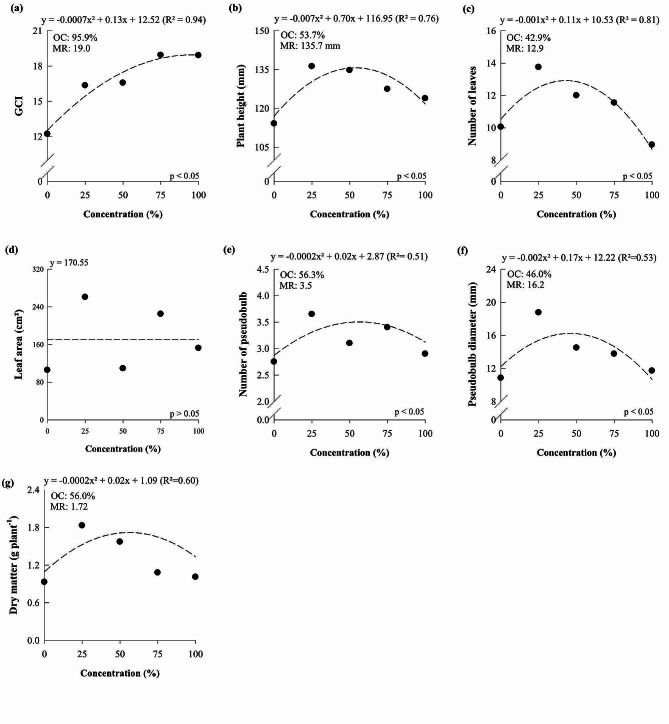
Green color index (**a**), plant height (**b**), number of leaves (**c**), leaf area (**d**), number of pseudobulbs (**e**), pseudobulb diameter (**f**), and dry matter of *Dendrobium Tubtim* Siam cultured under different nutrient solution concentrations. OC: optimal concentration; MR: maximum response

**Fig. 4:**
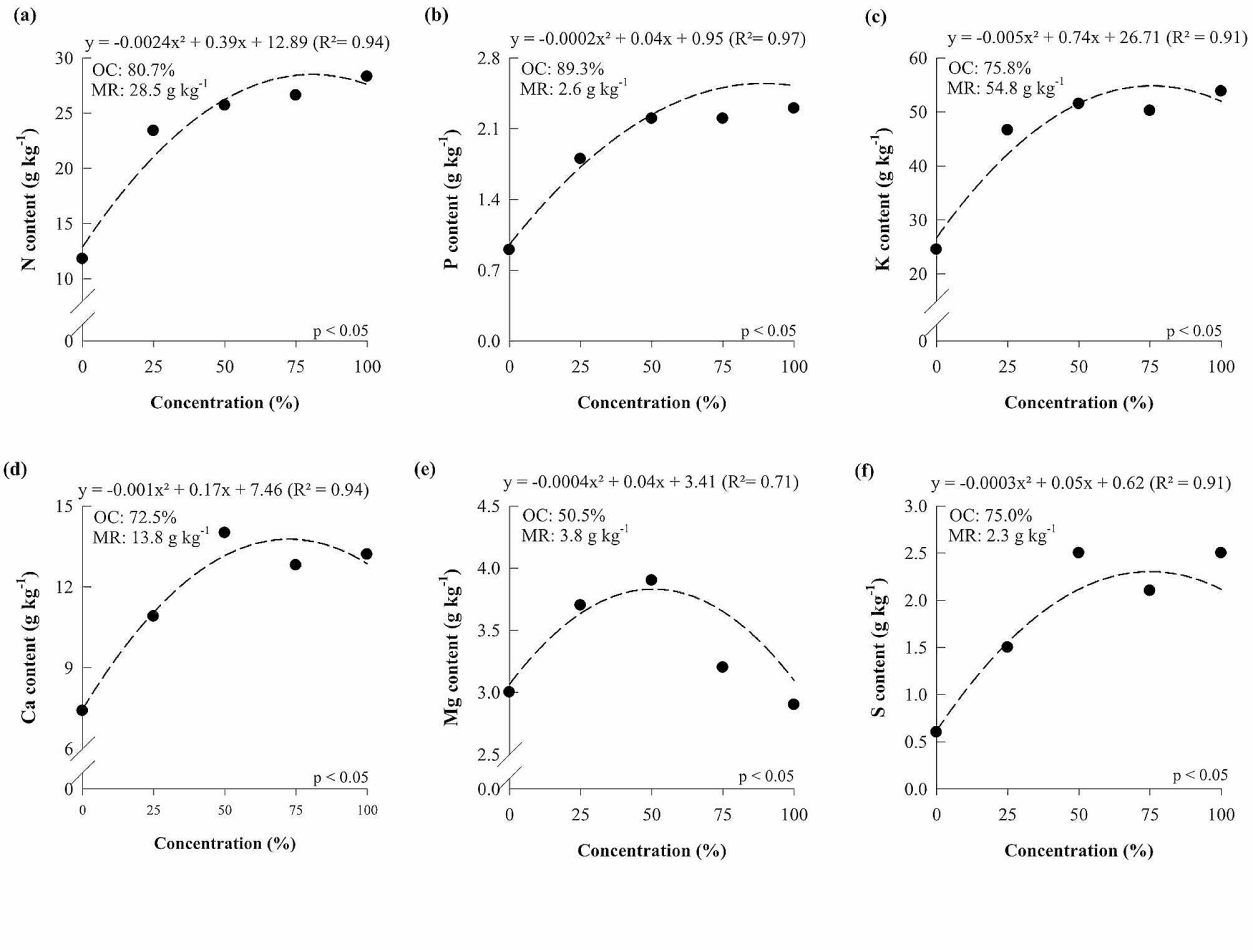
N (**a**), P (**b**), K (**c**), Ca (**d**), Mg (**e**), and S (**f**) contents of *Phalaenopsis Taisuco* Swan grown under different nutrient solution concentrations. OC: optimal concentration; MR: maximum response

**Fig. 5:**
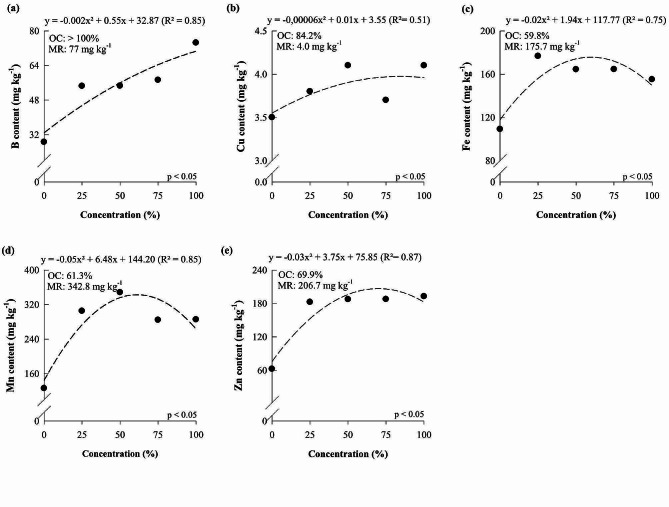
B (**a**), Cu (**b**), Fe (**c**), Mn (**d**), and Zn (**e**) contents of *Phalaenopsis Taisuco* Swan cultivated under different concentrations of nutrient solution. OC: optimal concentration; MR: maximum response

**Fig. 6:**
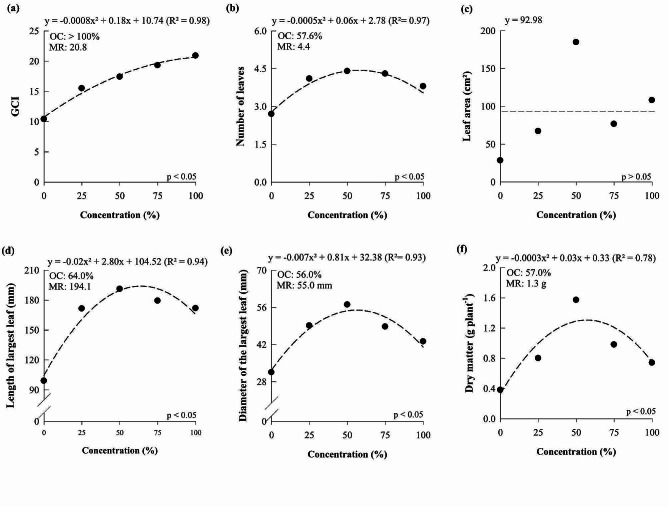
Green color index (**a**), number of leaves (**b**), leaf area (**c**), length of the largest leaf (**d**), diameter of the largest leaf (**e**), and plant dry matter (**f**) of *Phalaenopsis Taisuco* Swan grown under different nutrient solution concentrations. OC: optimal concentration; MR: maximum response

Studies carried out with orchids have been dedicated to understanding the effects of nutritional management on plant growth and development. Thus, studies have focused on evaluating isolated nutrients [4, 6, 8, 34], and little was understood regarding the impact of using different concentrations of nutrient solutions in orchid cultivation. A study conducted with orchids aimed to identify an ideal solution for the cultivation of *Laelia autumnalis* and *Paphiopedilum insigne*, however, they still indicated the ‘Hoagland and Arnon solution’ as ideal for the growth of both evaluated species [32]. Our results reveal that *D.* Tubtim Siam and *P.* Taisuco Swan respond positively to the increase in the concentration of the nutrient solution proposed by Hoagland and Arnon (Figs. 1, 2 and 4, and 5), consequently improving growth and dry matter production (Figs. 3 and 6) and showing the importance of nutritional management for these species. However, both orchid species showed maximum levels of nutrients in plants when exposed to concentrations of the nutrient solution proposed by Hoagland and Arnon [24] at around 56 and 57%. There was a reduction in nutrient levels in plants when reaching concentrations of 100% of the nutrient solution proposed by Hoagland and Arnon [24]. This phenomenon may lead to a nutritional imbalance, hindering the ability to adequately meet the nutritional requirements of the studied species.

*Dendrobium* Tubtim Siam indicated a positive response to nutrient content, growth, and dry matter production by increasing the nutrient solution concentration proposed by Hoagland and Arnon up to 62%. However, it was found that the maximum contents of N and B in the plant were reached at concentrations equal to 72 and 87% of the nutrient solution proposed by Hoagland and Arnon. These results show that *D.* Tubtim Siam have a greater demand for these nutrients compared to other nutrients, requiring an adjustment in the concentrations of these nutrients in the nutrient solution. B and N are two essential elements for the nutrition of orchids, as they participate in several physiological and biochemical processes in the plants [26, 35, 36]. A study conducted with different concentrations of nitrogen in the culture medium on the in vitro growth of four orchid species (*Cattleya loddigesii*, *Dendrobium nobile*, *Oncidium flexuosum*, and *Phalaenopsis amabilis*) also showed that nitrogen significantly influences plant growth [37].

In *P.* Taisuco Swan, there was an increase in nutrient content, growth, and dry matter production up to the average concentration of 77% of the nutrient solution proposed by Hoagland and Arnon. However, the maximum B content in the plant was reached with a nutrient solution concentration above 100%, indicating that this species has a greater demand for this nutrient. These results reinforce the need to adjust the nutrient concentrations in nutrient solutions for *P.* Taisuco Swan and *D.* Tubtim Siam, mainly for B.

The results showed that the two orchid species studied reached the maximum dry matter production of 1.72 and 1.31 g, which corresponded to the Hoagland and Arnon nutrient solution concentration equal to 56 and 57%, respectively (Figs. 3 and 5). These results show that the cultivation of orchids with nutrient solution concentrations above 56% can cause losses in dry matter production, possibly due to its antagonistic effect between nutrients. In *P.* Taisuco Swan, it was verified that P, K, Mg, and Mn contents and dry matter production reached the maximum in similar nutrient solution concentrations, showing that the increase of other nutrients in the solution start to limit the increase of P, K, Mg, and Mn in the plants, reflecting in dry matter loss. In *D.* Tubtim Siam, Mg was the most limited nutrient, as there was a decline in the content of this nutrient at concentrations above 51% of the nutrient solution (Fig. 4) which was possibly limited by the increase in the concentrations of other nutrients.

In this scenario, *P.* Taisuco Swan and *D.* Tubtim Siam showed maximum dry matter production at the concentration of the nutrient solution proposed by Hoagland and Arnon equal to 56 and 57%. However, it is evident that higher nutrient solution concentrations than those aforementioned would imply losses in dry matter production, with antagonistic effects between nutrients in the process of uptake by the plants. In this context, adopting a new nutrient solution capable of supplying these nutrients is necessary to provide a balanced nutrient supply in the vegetative growth phase of orchids. However, this is complex, as nutritional requirements vary between species, cultivars, reproductive stage, photoperiod, light intensity, temperature, and other factors [33]. . It is essential to understand the specific nutritional needs of each orchid to ensure its optimal growth and health [31, 38].

The optimal contents of nutrients in *P.* Taisuco Swan and *D.* Tubtim Siam associated with the general gas equation made it possible to propose a new nutrient solution (Table 1), allowing the proportion of nutrients in the nutrient solution to be the same as the one required by the plants. When calculating the nutrient concentration for the nutrient solution considering the optimum nutrient contents of plants, we found that N, P, Ca, and S concentrations are above the requirement of *P.* Taisuco Swan by 2.5, 25.0, 47.9, and 669.2%, respectively, while K, Mg, B, Cu, Fe, Mn, and Zn are below the requirement by 43.8, 27.8, 4.3, 73.7, 59.6, 88.6, and 98.5%, respectively, compared to the solution proposed by Hoagland and Arnon (Table 1). For *D.* Tubtim Siam, it was verified that P, Ca, Mg, and S are above the nutritional requirement of the species by 53.8, 83.2, 60.0, and 250.9%, respectively, while the concentrations of N, K, B, Cu, Fe, Mn and Zn are below the requirement by 7.4, 46.2, 18.3, 40.0, 28.4, 81.7, and 97.2%, respectively, in the nutrient concentration calculated for the nutrient solution compared to the solution proposed by Hoagland and Arnon (Table 1) .

It is important to highlight that among macronutrients, K was supplied below the necessary for the two species studied (Table 1), despite its maximum point in the concentration of the nutrient solution by Hoagland and Arnon being found at 51.8 and 75.8%. This result may be related to the excess Ca and Mg in the nutrient solution compared to the nutritional requirements of the plants. The antagonistic effect of Ca and Mg cations with K is well known in the literature, since the high concentration of one of these cations decreases the uptake of the other cations [26]. A study carried out with *Dendrobium nobile* Lindl verified a positive response of the plants as the K supply increased, relating the nutrient with the high vegetative growth of the plants [39]. A study conducted with *Ludisia discolor* found that the plants require a high concentration of K in the leaves and stems, low concentration in the roots, and furthermore, that K can aid in the metabolism of N and enhance the synthesis of amino acids and proteins [40].

K and N are the most required nutrients by orchids, showing the accumulation of 701.1 and 339.4 mg per plant in *D. nobile* Lindl [5], which highlights the need to adjust the concentration of these nutrients in the nutrient solution. The greater demand for these nutrients may be related to their mechanism of high accumulation of N, P, and K in the pseudobulbs, which act as a nutrient reservoir and may redistribute nutrients to young leaves under limiting conditions [19]. In natural environments, the association between orchids and microorganisms contributes to the nutrition of these plants, becoming an important source of nutrient reserves [12–14, 41].

Another crucial aspect is the significant disparity in sulfur (S) concentration between the Hoagland and Arnon solution and the calculated concentration. This discrepancy serves as justification for the absence of a suitable model for sulfur content in P. Taisuco Swan (Fig. 1f), where there is an excess supply of S well above the plant’s demand. With model adjustment, this discrepancy would be rectified. This shows that the demand for this nutrient was already met at the lowest concentration of the nutrient solution. Additionally, the high P concentration in the nutrient solution can reduce Zn uptake, as already evidenced in *P.* Taisuco Swan, indicating that high concentrations of P provoked Zn deficiency [42]. N:S, P:S, K:S, Ca:S, and Mg:S ratios increased in the solutions calculated for *P.* Taisuco Swan and *D.* Tubtim Siam when compared with the solution proposed by Hoagland and Arnon, which was induced by the lower S requirements of these plants (Table 2). On the other hand, it decreased N:K and P:K ratios, which was induced by the greater demand of K. These facts show similar responses from the two orchid species studied, indicating a greater requirement of K and a reduced requirement of S when compared with other cultivated species. For micronutrients in general, there was an increase in concentrations in the solution calculated from the optimal dry matter contents for all nutrients, with a reduction in the ratios between micronutrients (Table 3). Despite the supply below the nutrient demand for orchids, these plants develop special mechanisms to survive in limited nutritional conditions without showing nutritional symptoms [31].

In this scenario, the present research unveils the first nutrient solution indicated for the cultivation of *D.* Tubtim Siam and *P.* Taisuco Swan in the vegetative growth phase based on the nutrient content in the dry matter of these plants.

Our results show that the plants of *D.* Tubtim Siam and *P.* Taisuco Swan presented better growth, resulting in an increase in dry matter production with an increase of up to 56 and 57% in the concentration of the nutrient solution proposed by Hoagland and Arnon, respectively. Thus, the first hypothesis is partially supported by the data of this research, given the better performance of the plants in the vegetative growth phase in diluted concentrations of the nutrient solution. Furthermore, it was found that the adequate nutrient solution, calculated from the nutrient contents in the dry matter of orchid plants, was different from the nutrient solution proposed by Hoagland and Arnon [34], which is not consistent with the second hypothesis tested in our study. The results of this research will enable the cultivation of both orchid species using a more balanced nutrient solution for the vegetative growth phase, possibly helping to achieve optimal plant development and enhance the sustainable cultivation of these species worldwide.

## Methods

### Cultivation conditions and experimental design

Two experiments were carried out concurrently with plants of *Dendrobium* Tubtim Siam and *Phalaenopsis* Taisuco Swan at the São Paulo State University (Unesp), municipality of Jaboticabal, Brazil. The experiment was conducted in a completely randomized design with five treatments based on different concentrations of the nutrient solution proposed by Hoagland and Arnon [24] (0; 25; 50; 75 and 100%), with 20 biological replicates per treatment.

The experimental plots were characterized by plants grown in black polyethylene pots (upper diameter: 13 cm; lower diameter: 8.4 cm; height: 10.6 cm) with a volume of 0.9 dm^3^. The pots were filled with Sphagnum substrate (moss) and placed on suspended tables of 0.65 m in height. The seedlings were obtained from in vitro propagation (seeding), in a process from sowing to acclimatization that lasted 12 months. The acclimatization process was carried out in six months by removing the seedlings from the flasks and transplanting them in substrate (sphagnum). The plants were grown in a nursery with a transparent plastic cover and a sombrite® screen with 80% shading.

The pots, made of black polyethylene, provided a stable and well-drained environment for the plants, allowing them to thrive. Initially, after transplanting, a volume of 100 mL of the nutrient solution was applied to each pot. The pH of the solution was carefully adjusted to fall within the range of 5.5 to 6.5, optimizing nutrient availability and uptake by the plants. This initial application continued for a period of up to 150 days after transplanting. Following the initial application, a weekly regimen of nutrient solution application was implemented. Each week, a volume of 200 mL of the nutrient solution was administered to the pots. This regular application aimed to provide a consistent and sufficient supply of nutrients to support the plants’ growth and development. To prevent nutrient losses, special measures were taken. Cups were strategically placed beneath the pots to collect any leached portion of the nutrient solution. This prevented valuable nutrients from being lost and ensured that the plants received the intended amount of nutrients throughout the experiment. In addition to the nutrient solution application, irrigation was performed to provide adequate water supply to the plants. Two days after each nutrient solution application, irrigation was carried out twice a week during winter and three times a week during summer. Distilled water, in a volume of 100 mL per pot, was used for irrigation purposes.

### Experimental evaluations

Experimental evaluations in both experiments were performed at 330 days after transplanting, at the end of the vegetative growth phase.

#### Green color index

The evaluation of the green color index (GCI) was carried out using a portable device (portable chlorophyll meter, model CCM-200, OptiScience®) in the central part of the adaxial surface of the last fully developed leaf.

#### Plant growth

Plant height was measured with using a tape measure; pseudobulb diameter and the diameter and length of the largest leaf were measured using a digital caliper; and the number of leaves and number of pseudobulbs were measured using simple counting. Furthermore, the leaf area of all plant leaves was determined using a digital meter (Li-Cor, model L1-3000®).

#### Dry matter production

To determine dry matter production, first the plants were washed in deionized water, detergent solution (0.1% v/v), HCl solution (0.3% v/v), and deionized water for decontamination. Subsequently, the plants were placed in kraft paper bags and dried in an oven with forced air circulation (65 ± 5 ºC) until reaching constant mass. Then, dry matter was determined on a semi-analytical scale.

#### Chemical analysis in plant tissue

The plant material was ground after drying using a Wiley mill. Then, 0.1 g of plant material was weighed and subsequently digested through nitroperchloric digestion using a block digester. After samples were digested, the P content was determined by the colorimetric method with the using a spectrophotometer; the S content was determined by the turbidity method using barium chloride; and K, Ca, Mg, Cu, Fe, Mn, and Zn were determined by atomic absorption spectrophotometry [43, 44]. The N content was determined by the Kjeldahl method, in which the sample was digested with concentrated sulfuric acid under heating. Subsequently, the extract was alkalized with sodium hydroxide and the ammonia produced in the process was distilled and trapped in a boric acid indicator solution. Finally, titration with sulfuric acid (0.05 N) was carried out [43, 44]. The B content was determined by digesting the plant material using a muffle furnace. The content was determined by the spectrophotometric method using azomethine-H.

### Statistical analysis and adjustment of regression models

Data were organized in electronic spreadsheets and all statistical analyzes and adjustments of polynomial regression models were performed using the Python programming language (version 3.9.7; Python Software Foundation).

#### Preposition tests

The Shapiro-Wilk (*p* > 0.05) [45] and Levene’s homoscedasticity (*p* > 0.05) [46] tests were performed.

#### Analysis of variance and adjustment of regression models

Data were submitted to analysis of variance (*p* < 0.05) and regression models were adjusted by the F test (*p* < 0.05). Subsequently, the maximum point of the response variables and the nutrient solution concentration that provided the maximum point were calculated from the derivation of the adjusted models.

### Determination of nutrients concentrations in the nutrient solution

In order for the solution to meet the nutritional requirements of each orchid under study and for the nutrients to be depleted proportionally, their proportion in the nutrient solution must be the same as that required by the plants, that is, the one presented in the dry matter of leaves of well-nourished plants. The nutrient concentration for the nutrient solution was calculated proportionally to the nutrient concentrations in the dry matter of *D.* Tubtim Siam and *P.* Taisuco Swan, based on the methodology proposed by Martinez and Clemente [33]. The method is based on the nutrient concentration in the plant tissue and on the general gas equation.

#### General gas equation

To determine the total concentration of ions in the solution (n), the general gas equation was used, which is based on the atmospheric pressure (P), volume of solution (V), temperature (T), and the universal gas constant (R) (Eq. 1). For the present study, a solution temperature of 25º C, atmospheric pressure of 0.8 atm, and a volume of 1 L was considered, resulting in a number of of ions in solution of 32.73 mM.

1 $$ \text{P}\text{V}=\text{n}\text{R}\text{T}$$

#### Proportionality of nutrient contents in the plant tissue and nutrient solution

To calculate the proportionality of nutrient contents in the plant tissue and nutrient solution, the nutrient contents of the plants were transformed into mg/100 g of dry matter, transforming these values into mM. To calculate the nutrient concentration in the nutrient solution, the optimal content adjusted for each nutrient obtained in the cultivation of plants in the solution proposed by Hoagland and Arnon was considered [24]. Subsequently, the sum of the concentrations in mM of nutrients in the dry matter of *D.* Tubtim Siam and *P.* Taisuco Swan was performed, totaling 221.5 and 411.2 mM, respectively. To determine the individual concentration of nutrients, a multiplication constant was calculated from the ratio of the number of mmol of ions in solution, which was calculated by the general gas equation and the total concentration of ions in the plant tissue, finding the constants of 0.1478 and 0.0796 for *Dendrobium* Tubtim Siam and *Phalaenopsis* Taisuco Swan, respectively. Based on this constant, multiplication was carried out with the nutrient content in the plant tissue (mM), obtaining the ideal concentration of the nutrient for the nutrient solution of each orchid species. Additionally, the osmotic pressure of each nutrient solution was calculated from the volume (V), number of moles (ns), temperature, and the gas constant (R) (0.00832 Mpa K^-1^ mol at 273 K) (Eq. 2) [25].

2 $$ {\psi } \left(\text{M}\text{P}\text{a}\right)= -\text{n}\text{s}\text{R}\text{T}\text{V}$$

Finally, the electrical conductivity (EC) of the nutrient solution was calculated considering the total number of ions in solution and the constant of 0.0698 [25] (Eq. 3).

3 $$ \text{E}\text{C}\,\text{m}\text{S}\,{\text{c}\text{m}}^{-1}= \text{t}\text{o}\text{t}\text{a}\text{l}\,\text{i}\text{o}\text{n}\text{s} \left(\text{m}\text{M}\right) \times 0.0698$$

#### Ratio of nutrients in the nutrient solution

The nutrient concentration ratio in the nutrient solution was evaluated and the ratios of N:P, N:K, N:Ca, N:Mg, N:S, P:K, P:Ca, P:Mg, P: S, K:Ca, K:Mg, K:S, Ca:Mg, Ca:S, and Mg:S were determined for macronutrients and B:Cu, B:Fe, B:Mn, B:Zn, Cu:Fe, Cu :Mn, Cu:Zn, Fe:Mn, Fe:Zn, and Mn:Zn were determined for micronutrients.

### Electronic supplementary material

Below is the link to the electronic supplementary material.

Supplementary Material 1

Supplementary Material 2

## Data Availability

The datasets generated and/or analyzed during the current study are available from the corresponding author on reasonable request.
