# Supplementary figures and images for: Optimizing nutrient solution for vegetative growth of Dendrobium Tubtim Siam and Phalaenopsis Taisuco Swan through plant tissue nutrient balance estimation
Source: BMC Plant Biol. 2024 Apr 13;24:280. doi: 10.1186/s12870-024-04931-x (PMC11015735; doi:10.1186/s12870-024-04931-x)

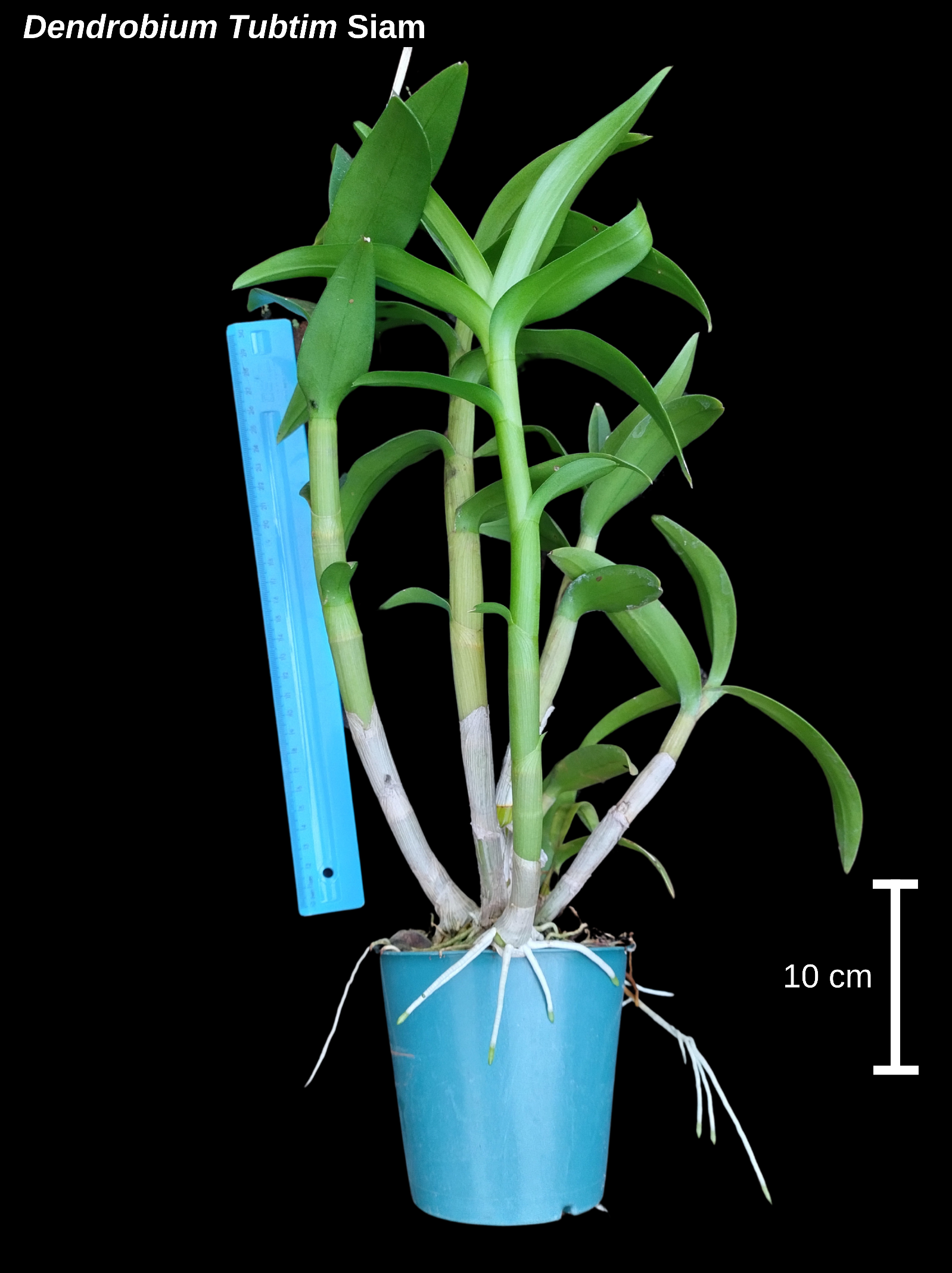


**Supplementary figure 1**. *Dendrobium Tubtim* Siam plants in optimal growth condition.

Supplement: Supplementary file 1 — Supplementary Material 1 [file 12870_2024_4931_MOESM1_ESM.docx]

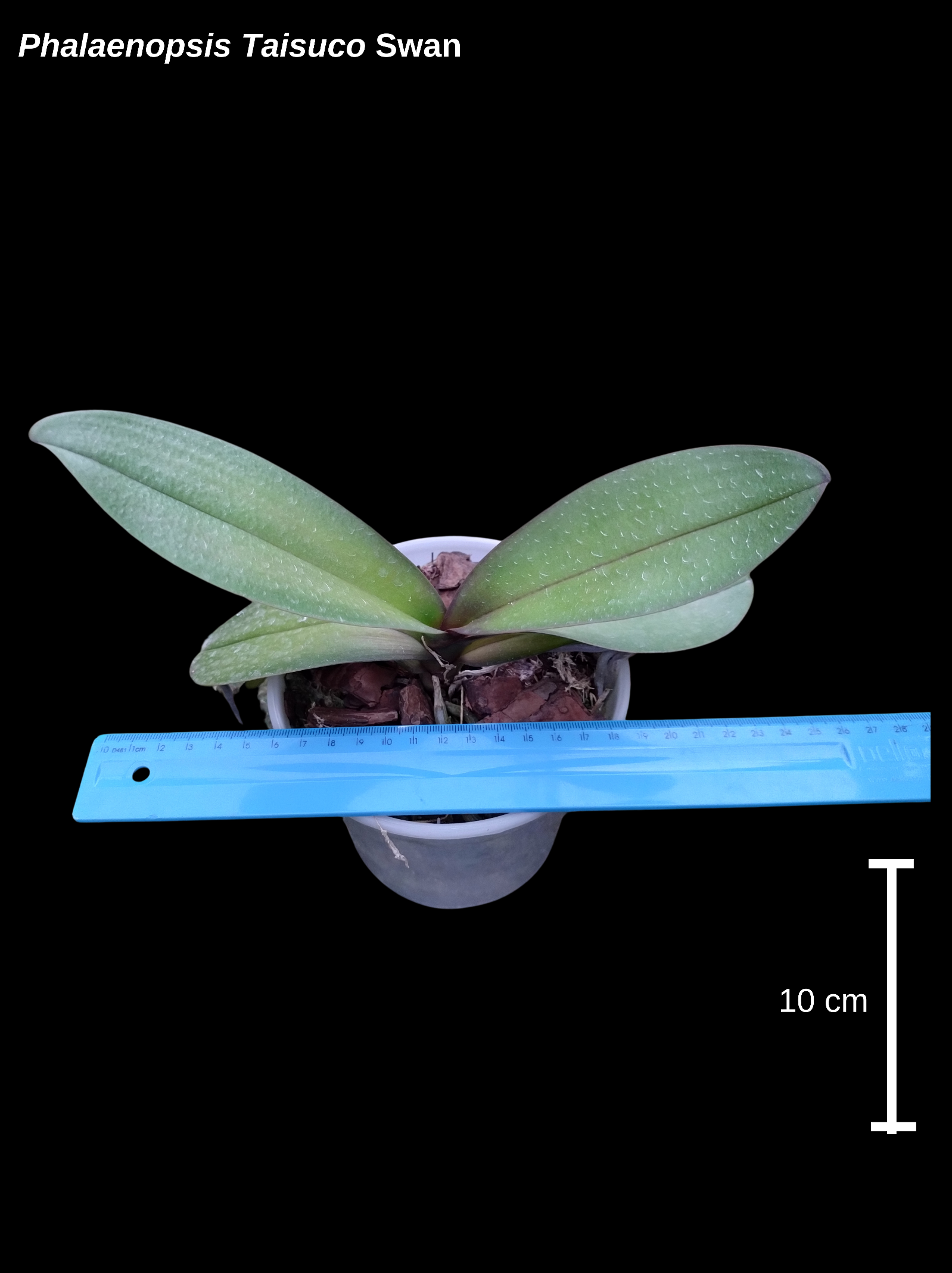


**Supplementary figure 2**. *Phalaenopsis Taisuco* Swan plants in optimal growth condition.

Supplement: Supplementary file 2 — Supplementary Material 2 [file 12870_2024_4931_MOESM2_ESM.docx]
